# Ductal carcinoma in situ: to treat or not to treat, that is the question

**DOI:** 10.1038/s41416-019-0478-6

**Published:** 2019-07-09

**Authors:** Maartje van Seijen, Esther H. Lips, Alastair M. Thompson, Serena Nik-Zainal, Andrew Futreal, E. Shelley Hwang, Ellen Verschuur, Joanna Lane, Jos Jonkers, Daniel W. Rea, Jelle Wesseling, on behalf of the PRECISION team

**Affiliations:** 1 Division of Molecular Pathology, The Netherlands Cancer Institute, Amsterdam, The Netherlands; 2 Dan L Duncan Comprehensive Cancer Center, Baylor College of Medicine, Houston, TX USA; 3 Department of Medical Genetics, University of Cambridge, Cambridge, UK; 4 Department of Surgery, University of Texas MD Anderson Cancer Center, Houston, TX USA; 5 Department of Surgery, Duke University Comprehensive Cancer Center, Durham, NC USA; 6 Borstkankervereniging Nederland, Utrecht, The Netherlands; 7 Health Cluster Net, Amsterdam, The Netherlands; 8 Oncode Institute, Amsterdam, The Netherlands; 9 Department of Medical Oncology, University of Birmingham, Birmingham, UK; 10 Department of Pathology, The Netherlands Cancer Institute, Amsterdam, The Netherlands; 11 Department of Pathology, Leiden University Medical Center, Leiden, The Netherlands

**Keywords:** Prognostic markers, Breast cancer

## Abstract

Ductal carcinoma in situ (DCIS) now represents 20–25% of all ‘breast cancers’ consequent upon detection by population-based breast cancer screening programmes. Currently, all DCIS lesions are treated, and treatment comprises either mastectomy or breast-conserving surgery supplemented with radiotherapy. However, most DCIS lesions remain indolent. Difficulty in discerning harmless lesions from potentially invasive ones can lead to overtreatment of this condition in many patients. To counter overtreatment and to transform clinical practice, a global, comprehensive and multidisciplinary collaboration is required. Here we review the incidence of DCIS, the perception of risk for developing invasive breast cancer, the current treatment options and the known molecular aspects of progression. Further research is needed to gain new insights for improved diagnosis and management of DCIS, and this is integrated in the PRECISION (PREvent ductal Carcinoma In Situ Invasive Overtreatment Now) initiative. This international effort will seek to determine which DCISs require treatment and prevent the consequences of overtreatment on the lives of many women affected by DCIS.

## Background

Ductal carcinoma in situ (DCIS) was rarely diagnosed before the advent of breast screening, yet it now accounts for 25% of detected ‘breast cancers’. Over 60,000 women are diagnosed with DCIS each year in the USA,^1,2^ >7000 in the UK^3^ and >2500 in the Netherlands.^4^ DCIS is a proliferation of neoplastic luminal cells that are confined to the ductolobular system of the breast. If DCIS progresses to invasive breast cancer, DCIS cells penetrate the ductal basement membrane and invade the surrounding parenchyma. Individual lesions differ in aspects of the disease: presentation, histology, progression, and genetic features.^5,6^ Despite being pre- or non-invasive, DCIS is often regarded as an early form of (Stage 0) breast cancer. Therefore, conventional management includes mastectomy or breast-conserving surgery supplemented with radiotherapy; in some countries, adjuvant endocrine therapy is added. Regrettably, current therapeutic approaches result in overtreatment of some women with DCIS (Box 1). The Marmot Report in 2012 recognised the burden of overtreatment to women’s wellbeing.^7^ In effect, women with DCIS are labelled as ‘cancer patients’, with concomitant anxiety and negative impact on their lives, despite the fact that most DCIS lesions will probably never progress to invasive breast cancer. Owing to the uncertainty regarding which lesions run the risk of progression to invasive cancer, current risk perceptions are misleading and consequently bias the dialogue between clinicians and women diagnosed with DCIS, resulting in overtreatment for some, and potentially many, women.

Improving the management and treatment of DCIS presents a central challenge: distinguishing indolent, harmless DCIS lesions from potentially hazardous ones. This poses a fundamental question to address: ‘Is cancer always cancer?’. To answer this question, we need to adopt an interdisciplinary and translational approach, merging fields of epidemiology, molecular biology, clinical research and psychosocial studies. How low does the risk need to be to refrain from treating DCIS? What are the prognostic markers and read-outs we can rely on? How do we frame and communicate the risks involved?

In this review, we describe the current approaches to diagnosing DCIS, the perception of the risk of developing invasive breast carcinoma, the treatment options available following a diagnosis and a current knowledge of the progression of DCIS, before outlining future endeavours and the need for an integrated approach that blends clinical and patient insights with scientific advances.

Box 1: Consequences of overdiagnosis in DCIS: Impact of DCIS on a woman’s life The diagnosis of DCIS labels women as being at risk for invasive breast cancer. Despite the good prognosis and normal life-expectancy, women diagnosed with DCIS may experience substantial psychological distress^29^ and overestimate the implications of a DCIS diagnosis.^34,35,92^ Comorbidity of surgery and prior depression have been reported as important factors related to worse quality of life in these women.^29^ Critical questions yet to be answered include: (i) Can the way in which a diagnosis for DCIS is communicated be improved? (ii) Can the labelling effects of a diagnosis of DCIS be mitigated, while ensuring adequate follow-up of these high-risk women? And, finally, (iii) what is the impact on quality of life for active surveillance of women diagnosed with low-grade DCIS? Addressing these questions requires central involvement of patient voices to improve clarity not only for patients but also for healthcare providers about the implications and risks of a diagnosis of DCIS.^93^

## DCIS incidence

The number of women diagnosed with DCIS over the past few decades largely follows the introduction of population-based breast cancer screening.^8–12^ The European standardised rate of in situ lesions has increased four-fold, from 4.90 per 100,000 women in 1989 (accounting for 4.5% of all diagnoses registered as breast cancer) to 20.68 in 2011 (accounting for 12.8% of all diagnoses registered as breast cancer; www.cijfersoverkanker.nl). Of all in situ breast lesions reported, 80% are DCIS.^12,13^ Nevertheless, the incidence of mortality from early-stage breast cancer has not decreased concurrently with DCIS detection and treatment, indicating that managing DCIS does not reduce breast-cancer-specific mortality and therefore could be considered as overtreatment.^8,11^ A review of autopsies in women of all ages revealed a median prevalence of 8.9% (range 0–14.7%). For woman aged >40 years, this prevalence was 7–39%,^14^ whereas breast cancer is diagnosed in only 1% of women in the same age range.^13^ These data suggest that a large number of women might have an undetected source of DCIS that will never become symptomatic.

## Current diagnosis and imaging

DCIS is usually straightforward to detect by mammography because of its association with calcifications; the proliferation of cells itself is not visible on the mammogram. However, as only 75% of all DCIS lesions contain calcifications,^15^ a substantial percentage of DCIS lesions will not be detected by mammography, implying that some lesions might be mammographically occult or that the diameter of the area containing calcifications underestimates the extent of DCIS.^16,17^ This suggests that DCIS might be left behind following breast-conserving treatment in a proportion of cases.

After detection, the lesion is classified by the pathologist by histological features as low, medium or high grade, which is assumed to correspond to the level of aggressiveness. Surprisingly, many grading systems exist.^18^ An agreement on classification was reached during a consensus meeting in the USA where consensus was reached to include nuclear grade, presence of necrosis, cell polarisation and architectural patterns in the pathology report.^19,20^ Some studies showed a slight tendency for high-grade DCIS to progress to invasive breast cancer,^21^ but others demonstrated that grade is not significantly associated with the risk of local invasive recurrence.^22,23^ Greater consistency in grading could result in more certainty about the association of morphology with progression and outcome. In addition, as grade is not a perfect discriminator for progression risk, other risk discriminators, such as molecular biomarkers, are examined (discussed later in ‘Molecular, cellular and microenvironmental aspects’).

## Perception of risk

Generally, patients diagnosed with DCIS have an excellent long-term breast-cancer-specific survival of around 98% after 10 years of follow-up^24–27^ and a normal life expectancy.^27^ However, a consensus in the medical community is lacking on how to effectively communicate to patients about DCIS and the associated risk of development into invasive cancer.^28^ It is essential to be aware of the fact that if the lower-grade DCIS (considered as the lower-risk lesions) progresses into invasive breast cancer, this will often be the lower-grade, slow-growing and early-detectable invasive disease, with excellent prognosis.

Because both diagnosis and treatment of the condition can have a profound psychosocial impact on a woman’s life, adequate perception of risk by both health professionals and patients is important in determining the appropriate modalities of treatment. Despite an excellent prognosis and normal life-expectancy, women diagnosed with DCIS experience stress and anxiety.^29^ Studies report that most women with DCIS (and early-stage breast cancer) have little knowledge and inaccurate perceptions of the risk of disease progression, and this misperception is associated with psychological distress.^30–36^ Women with DCIS make substantial changes to their behaviour after diagnosis, including smoking cessation and decreasing the use of postmenopausal hormones.^37^

Similar to progression rates for DCIS, classic lobular carcinoma in situ (LCIS) confers a risk of 1–2% per year to develop into invasive disease.^38,39^ First-line treatment for LCIS usually comprises active surveillance; unlike DCIS, doctors and patients accept the concept of active surveillance to monitor for progression of LCIS before administering any aggressive treatment. The need for effective doctor–patient communication is therefore essential for patients to understand the risk of recurrence.^40,41^ According to Kim et al.,^36^ women in whom DCIS was detected experienced high decisional conflict in treatment options and were not satisfied with the information provided to them. The development of a prediction tool could help to classify patients into risk groups and provide accurate guidance to patients, as well as healthcare professionals, in their choice of an appropriate treatment option.^42^ Nowadays, such a tool is even more important, as patients increasingly wish to engage in shared decision making about their disease.

## Treatment of DCIS

### Surgery and radiation therapy

Currently, breast-conserving treatment for DCIS is frequently recommended. A mastectomy is advised if the DCIS is too extensive to allow breast conservation.^43^ According to Thompson et al.,^21^ the recurrence rates (for both invasive and in situ) with 5 years median follow-up are 0.8% after mastectomy, 4.1% after breast-conserving surgery followed by radiotherapy and 7.2% after breast-conserving surgery alone. According to Elshof et al.,^22^ invasive recurrence rates are 1.9, 8.8 and 15.4%, respectively, after 10 years median follow-up. The 15-year cumulative incidence in the National Surgical Adjuvant Breast and Bowel Project 17 (NSABP17) trial of patients with clear margins is 19.4% after breast-conserving surgery alone and 8.9% after breast-conserving surgery followed by radiotherapy.^44^ Four randomised clinical trials have been performed to investigate the role of radiotherapy in breast-conserving treatment for DCIS after complete local excision of the lesion. In a meta-analysis, these trials show a 50% reduction in the risk of local recurrences (for both in situ and invasive) after radiotherapy.^45^ Radiotherapy was reported to be effective in reducing the risk of local recurrence in all analysed subgroups according to age, clinical presentation, grade and type of DCIS.

Adding radiotherapy to breast-conserving treatment reduces local recurrence rates but does not influence overall survival or breast-cancer-specific survival.^27,45,46^ The added value of conducting a sentinel node biopsy procedure is uncertain. In general, such a procedure is done with mastectomy for DCIS (since there is no opportunity to perform a subsequent sentinel node biopsy) or where there is a high suspicion for invasive disease even where DCIS alone is present in the preoperative biopsy.^47,48^

A recent study based on an analysis of data from the American Cancer Registry of >100,000 women diagnosed with DCIS suggests that aggressive treatment might not be necessary to save lives.^24,49^ A retrospective Surveillance, Epidemiology, and End Results (SEER) study demonstrated for the first time that patients with low-grade DCIS had the same overall survival and breast-cancer-specific survival rates with or without surgery.^49^ These findings prompted the breast healthcare community to explore innovative studies that could circumvent the need for harsh therapeutic intervention for treating an indolent condition.^24,49^

### Endocrine therapy

Owing to the side effects of hormonal therapy and ambiguous results from clinical trials, postmenopausal women with DCIS are rarely treated with endocrine therapy in many countries. In addition, the notion of systemic treatment for a localised disease with an excellent outcome is perceived as being counterintuitive.^21,50^ Two randomised clinical trials have investigated the role of tamoxifen – a drug that inhibits the oestrogen receptor (ER) – versus placebo in DCIS.^44,51^ The risk of subsequent invasive ipsilateral breast cancer was found to be reduced by tamoxifen in the NSABP trial^44^; the UK, Australia and New Zealand (UK/ANZ) DCIS trial demonstrated a reduction in recurrent DCIS but not in invasive breast cancer.^51^ Tamoxifen administration did not influence overall survival in either trial^52^ and appeared to be more effective at reducing the incidence of new breast events in patients who did not receive radiotherapy in the NSABP trial.^51^ Yet, a non-significant reduction in the incidence of new breast events was seen in the prospective series from the UK, independent of whether the patients received radiotherapy or not.^53^ Furthermore, to prevent one recurrence, 15 patients would need to be treated (the number needed to treat).^52^ In terms of efficacy, tamoxifen and anastrozole (an aromatase inhibitor) are comparable, and the percentage of women who reported side effects were 91% and 93% for anastrozole and tamoxifen, respectively. Although anastrozole administration more often causes side effects such as musculoskeletal pain, hypercholesterolaemia and strokes, tamoxifen is associated with muscle spasm, deep vein thrombosis and the development of gynaecological symptoms and gynaecological cancers.^54^ In the USA, the uptake of endocrine treatment is higher than in other countries, and nearly half of all ER positive patients are treated by additional adjuvant tamoxifen treatment, indicating a lack of consensus on the added value of this treatment.^55^

### Active surveillance

To address the question whether some patients with DCIS are overtreated, a group of patients not treated with conventional therapies should be studied. A prospective study with long-term follow-up is the only way to gain confidence regarding the natural course of DCIS, and therefore the potential need for interventions. Recently, three clinical trials (LORIS (United Kingdom, NCT02766881),^56^ COMET (United States of America, NCT02926911)^57,58^ and LORD (The Netherlands, NCT02492607))^59^ have opened to randomise patients with low-risk DCIS between active surveillance and standard treatment. Lower grades of DCIS are enrolled (grade 1 and/or grade 2 with limitations depending on the trial). Patients receive annual mammography (in COMET biannual mammography) in the active surveillance arm to monitor the lesions. Patients in the control arm will get conventional treatment (surgery often supplemented with radiotherapy). The primary outcome assesses whether active surveillance is non-inferior to surgery in terms of ipsilateral invasive breast-cancer-free survival^56^ (LORIS), ipsilateral invasive breast-cancer-free percentage at 2 years (COMET)^57^ or at 10 years (LORD).^59^ Because the primary outcomes of the trials are based on the occurrence of invasive disease during follow-up, it is essential to exclude an invasive component at the time of enrolment. Missed invasive disease at DCIS diagnosis is reported up to 26%.^60^ However, Grimm et al. found that, among trial-eligible patients, there was upstaging of 6, 7 and 10% for COMET, LORIS and LORD trials, respectively, compared with a general upstaging of 17% at the time of surgery for preoperatively diagnosed DCIS of all types.^61^ All trials include only pure DCIS with the use of multiple biopsies, additional biopsies in extended lesions and vacuum-assisted (large volume) biopsies.

## From DCIS to invasive breast cancer

### Proposed mechanisms for the development of invasive breast cancer

**Fig. 1:**
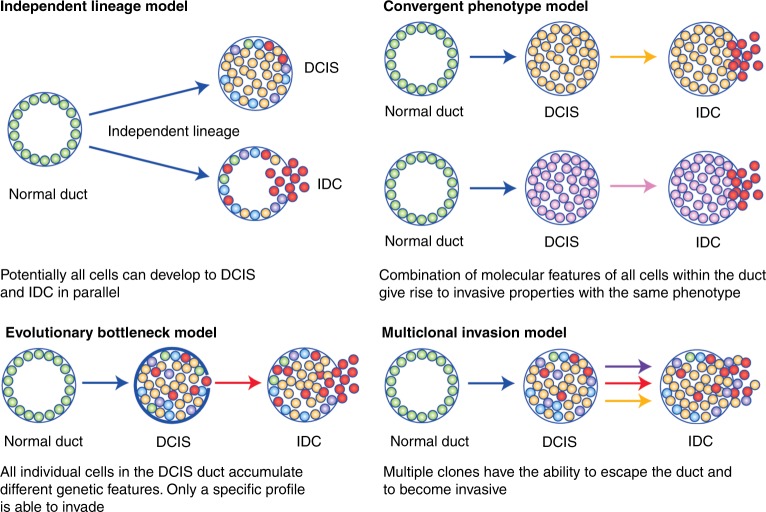
Overview of models showing four different theories of progression from ductal carcinoma in situ to invasive breast cancer

Although the natural course of the intraductal process is unknown, DCIS is considered to be a non-obligate precursor of invasive breast cancer. Four evolutionary models have been proposed to describe the progression of DCIS into invasive breast cancer (Fig. 1).

The first model is the **independent lineage model**. On the basis of mathematical simulations of the observed frequencies of the histological grade of DCIS and the histological grade of invasive disease in the same biopsy sample, Sontag et al. proposed that in situ and invasive cell populations arise from different cell lineages and develop in parallel and independently of each other.^62–64^ In support of this theory, Narod et al.^65^ state that small clusters of cancer cells with metastatic ability spread concomitantly through various routes to different organs and can therefore give rise to DCIS, invasive breast cancer and metastatic deposits simultaneously. Recent studies elucidating molecular differences between DCIS and invasive breast cancer further support the relevance of this model.^66^

The **convergent phenotype** **model** proposes that different genotypes of DCIS could lead to invasive breast cancer of the same phenotype. Furthermore, this model assumes that all the cells within the DCIS duct have the same genetic aberrations but that the combination of aberrations could differ between ducts (within the same DCIS lesion).^67,68^ Hernandez et al. demonstrated similarity in the genomic profiles of DCIS and invasive breast cancer in the majority of the matched pairs. However, in some cases, DCIS and adjacent invasive breast cancer differ in copy number and gene mutations, supporting the notion that, at least in some cases, progression is driven by specific clones leading to the same phenotype.^69^

In the **evolutionary bottleneck model**, individual cells within a duct are considered to accumulate different genetic aberrations; however, only a subpopulation of cells with a specific genetic profile is able to overcome an evolutionary bottleneck and invade into the adjacent tissue.^63,64,68^ This bottleneck model is supported by studies that report high genetic concordance between in situ and invasive lesions in addition to some differences between DCIS and invasive disease.^70^

In the **multiclonal invasion model**, multiple clones have the ability to escape from the ducts and co-migrate into the adjacent tissues to establish invasive carcinomas^63,64^ Casasent et al. demonstrated, using single-cell sequencing, that most mutations and copy number aberrations evolved within the ducts prior to the process of invasion. Shifts in clonal frequencies were observed, suggesting that some genotypes are more invasive than others. The same subclones were present in both in situ and in invasive regions with no additional copy number aberrations acquired during invasion and few invasion-specific mutations. These findings are, however, limited by their small sample size and comparison of contemporaneous DCIS and invasive disease.^63^

These putative models illustrate the potential complexity of the invasion process in DCIS and indicate that indolent lesions might become invasive via a combination of more than one of the proposed mechanisms.^6^

### Molecular, cellular and microenvironmental aspects

Many studies have focussed on identifying molecular markers of the invasive process and recent studies^69–72^ have linked mutations in *PIK3CA*, *TP53* and *GATA3* genes with aggressive DCIS; *TP53* mutations were reported to be exclusively associated with high-grade DCIS.^71,72^ However, the requirement for fresh tissue and large amounts of DNA for whole-exome or genome sequencing has limited the extent of studies for determining the landscape of genetic mutations in DCIS.

Some molecular analyses have shown that pre-invasive lesions and invasive breast cancer display remarkably similar patterns,^73–76^ indicating a common ancestor^77^; other groups have found that progression from DCIS to invasive breast cancer might be driven by a subset of cells with specific genetic aberrations, implying contribution to tumour initiation.^66,77–80^ PAM50 is a gene signature that can classify invasive breast cancer into five intrinsic subtypes (luminal A, luminal B, HER2-enriched, basal-like and normal-like), which adds prognostic and predictive information.^81^ Lesurf et al.^74^ applied the PAM50 signatures to DCIS and showed substantial differences between the subtypes, indicating that each PAM50 subtype undergoes a distinct evolutionary course of disease progression. Strikingly, their results showed that these properties, specific for the PAM50 subtypes, reflect changes that involve the microenvironment rather than molecular changes specific for epithelial cells. This supports increasing evidence for the role of the microenvironment in tumour progression and disease outcome more generally.^74^ Alcazar et al.^82^ demonstrated a switch to a less active tumour immune environment during the in situ to invasive breast carcinoma transition and identified immune regulators and genomic alterations that shape tumour evolution. Their data suggest that the levels of activated CD8+ T cells might predict which DCIS is likely to progress to invasive disease.^82^ In patients with invasive breast cancer – particularly those with triple-negative and HER2-positive subtypes – the presence of tumour-infiltrating lymphocytes (TILs), especially higher numbers of CD8+ cells, together with fewer FOXP3+ regulatory T cells, is associated with a better outcome.^83^

One of the key molecular differences between DCIS and invasive breast cancer is the prevalence of HER2 amplification: 34% for DCIS^84^ versus 13% for invasive disease.^85^ HER2 amplification might be a prognostic factor in predicting an in situ recurrence after DCIS, but it seems not to be predictive for an invasive recurrence.^86^ That said, one study with a long follow-up (mean follow-up >15 years) counterintuitively demonstrated that HER2 positivity in primary DCIS was associated with a lower risk of late invasive breast cancer compared with HER2 negativity.^87^ In HER2-positive DCIS, TILs are present at higher levels, but an association with an invasive recurrence risk after DCIS has not been reported.

A caveat of molecular studies on DCIS is the fact that most studies examine relatively small series of DCIS lesions with a contemporaneously adjacent invasive component, instead of a metachronous (subsequent) invasive lesion developing during follow-up. Thus these series are inherently biased, because the majority of the DCIS lesions will never develop an invasive component. In addition, most studies do not distinguish between in situ or invasive recurrences after DCIS. Two biomarker-based assays have been developed for DCIS,^88,89^ which purport to predict the benefit of radiotherapy for DCIS. However, the assays only discriminate between the risk of an in situ versus an invasive recurrence after DCIS to a limited extent. This difference is important for the women involved, especially regarding treatment choices, prognosis and psychosocial impact. Furthermore, intratumoural heterogeneity complicates our understanding of the relationship between DCIS and its invasive counterpart, as most studies only analyse a small proportion of an often heterogeneous lesion or analyse a bulk tissue sample in which small cell populations are easily overlooked.^64^ The low number of samples and lack of longitudinal follow-up data mean that our overall molecular knowledge of the landscape of changes in DCIS is limited.

#### Looking ahead

Uncertainty exists about how DCIS develops, and global consensus is lacking as to how best to optimally manage this disease. A better understanding of the biology of DCIS and the natural course of the disease is required to support patients and healthcare professionals in making more informed treatment decisions, in turn reducing the current overtreatment of DCIS. In 2014, Gierisch et al.^90^ described and prioritised knowledge gaps of patients and decision makers with regards to future research of DCIS for the Patient-Centered Outcomes Research Institute (PCORI), a private, non-governmental, non-profit, USA-based institute created by The Patient Protection and Affordable Care Act of 2010 to ‘help people make informed healthcare decisions, and improve healthcare delivery and outcomes’. By reviewing the existing literature and using a forced-ranking prioritisation method, a list of ten evidence gaps was created (Table 1). Issues that needed immediate attention include the effective communication of information about diagnosis and prognosis and dedicated efforts to fill the knowledge gaps regarding long-term implications and risks of a diagnosis of DCIS.^90^

**Table 1:** How PRECISION addresses research needs for DCIS management (adapted from Gierisch et al.^90^)

Rank	Prioritization of research need according to Gierisch et al.^90^	Recommended study design by Gierisch et al.^90^	Addressed in PRECISION
1	Validate risk-stratification models	Meta-analysis or individual patient data analysis across RCTs or observational study using existing data sources	Combining retrospective case-control studies based on nationwide, population-based cohorts
2	Compare safety and effectiveness of a management strategy involving no immediate treatment (i.e. monitoring/observation/active surveillance) versus immediate treatment with surgery, RT, and/or medical therapy	Prospective observational study	Prospective RCT to test safety of active surveillance for low-grade DCIS
3	Determine whether safety and effectiveness of DCIS management strategies differ depending on variations in clinical, pathologic, and genomic presentations of DCIS	Meta-analysis or individual patient data analysis across RCTs or observational study using existing data sources	Combining results from retrospective case-control studies and prospective RCTs
4	Comparative effectiveness of different approaches to communicating the diagnosis of DCIS to the patient	RCT	Evaluation level of being informed, QoL, and HTA in prospective RCTs
5	Comparative effectiveness of decision-making tools compared with usual care	RCT	Evaluation of prognostic factors, QoL, and HTA in prospective RCTs
6	Comparative sensitivity and specificity of breast MRI, mammography, and other preoperative imaging evaluations for detecting occult invasive breast cancer	Observational study either collecting new data or using existing data sources	Analysis based on mammograms collected in prospective RCTs
7	Assess effect of DCIS management strategies on comorbid conditions	RCT	Prospective RCTs
8	Compare safety and effectiveness of partial-breast RT versus whole-breast RT	RCT	Not addressed in this research proposed
9	Identify most important patient-centered outcomes for women diagnosed with DCIS	Observational study requiring new data collection	Prospective RCT for patient-centred outcomes
10	Assess effect of DCIS management strategies on rates of invasive cancer	Observational data using existing data	Retrospective case-control studies and prospective RCTs

This Table was adapted from Annals of Internal Medicine, Gierisch, J.M., Myers, E.R., Schmit, K.M., Crowley, M.J., McCrory, D.C., Chatterjee, R., Coeytaux, R.R., Kendrick, A. and Sanders, G.D., Prioritization of Research Addressing Management Strategies for Ductal Carcinoma In Situ, Volume 160, Issue 7, Pages 484-491. Copyright © 2014 American College of Physicians. All Rights Reserved. Reprinted with the permission of American College of Physicians, Inc. *DCIS* ductal carcinoma in situ, *RT* radiotherapy; *RCT* randomised controlled trial; *QoL* quality of life, *HTA* health technology assessment, *MRI* magnetic resonance imaging, *PRECISION* PREvent ductal Carcinoma In Situ Invasive Overtreatment Now

To address these priorities in DCIS, a multidisciplinary approach with scientific, clinical and patient expertise is needed. Data from large retrospective cohorts should be integrated with in vitro and in vivo studies and the results should be validated to transform clinical practise. To fund such a large multinational consortium, Cancer Research UK and the Dutch Cancer Society (KWF) partnered to support the Grand Challenge^91^ award in 2017, the PREvent ductal Carcinoma In Situ Invasive Overtreatment Now (PRECISION) initiative (see Box 2 and Supplementary Material for more information about PRECISION).

Box 2: The PRECISION initiative The general aim of the CRUK/KWF Grand Challenge PRECISION Initiative (www.dcisprecision.org) is to prevent the burden of DCIS overtreatment. ‘PRECISION’ is the acronym for ‘PREvent ductal Carinoma In Situ Invasive Overtreatment Now’. PRECISION ultimately aims to develop novel tests that promote informed and shared decision-making between patients and clinicians, without comprising the excellent outcomes for DCIS management that are presently achieved. The PRECISION initiative consists of seven interlinked work packages (WPs). WP1 enables the collection of large tissue resources. These series will be used in WP2–4 for genomic characterisation to find key drivers (WP2), characterising the function of the microenvironment in DCIS biology (WP3), and the role of imaging in DCIS prognosis and outcome (WP4). WP5 comprises functional validation of the key drivers in in vitro and in vivo models and WP6 will incorporate all the information obtained in a clinical risk prediction model. The three prospective studies will be used for overall validation through collection of blood and tissue samples (WP7). Importantly, patient advocates are actively involved in every part of the project. Ultimately, all these efforts may contribute to a more balanced perception of risk regarding non-life-threatening precancerous lesions in general, reducing anxiety, and preserving quality of life.

## Conclusion

Current perceptions of the risk-framing dialogue between clinicians and women diagnosed with DCIS are currently resulting in the overdiagnosis and overtreatment of DCIS. The need to reframe perceptions of risk and to avoid overtreatment is urgent, as overtreatment leads to physical and emotional harm for patients and to unnecessary costs for society. Specifically, knowing when a lesion could be or will not be life-threatening requires a thorough understanding of the progression and evolution of DCIS. To this end, initiatives, such as PRECISION, have been set out to reduce the burden of overtreatment of DCIS by gaining deep knowledge about the biology of DCIS. This knowledge will contribute to informed decision-making between patients and clinicians, without compromising the excellent outcomes for DCIS that are presently achieved. Dealing with this challenge demands an integrated approach that blends clinical and patient insights with scientific advances in order to improve the diagnosis, treatment and management of DCIS. To accomplish this, it is critical that patient advocates, scientists and clinicians work together, exemplified by a collaborative patient advocate and scientist in the PRECISION research team video**:** https://youtu.be/aoGSDDto1Gc.

## Supplementary information

PRECISION initiative

## Data Availability

Not applicable.
