# Supplementary material for: Ductal carcinoma in situ: to treat or not to treat, that is the question
Source: Br J Cancer. 2019 Jul 9;121(4):285–92. doi: 10.1038/s41416-019-0478-6 (PMC6697179; doi:10.1038/s41416-019-0478-6)
Supplement: Supplementary file 1 — PRECISION initiative [file 41416_2019_478_MOESM1_ESM.docx]

Supplementary Information

**PREvent ductal Carcinoma In Situ Invasive Overtreatment Now (PRECISION) initiative**

In 2015, we commenced the PREvent ductal Carcinoma In Situ Invasive Overtreatment Now (PRECISION) initiative by applying to the Cancer Research UK Grand Challenge theme ‘When is cancer not really cancer’. We brought together a complementary team of scientific, clinical and patient expertise needed to change clinical practice regarding the diagnosis and management of DCIS, to ignite new ideas and to hasten the translation of science to the clinical setting. Through this multidisciplinary approach we are aiming to address 9 out of the top 10 priorities in DCIS research as outlined by Gierisch *et al.*^90^ (Table 1). In 2017, the PRECISION initiative was awarded £15 million to distinguish harmless from hazardous DCIS. The PRECISION initiative consists of seven interlinked work packages and four supporting working groups, as outlined in Supplementary Figure 1.

**Curation of DCIS cohorts (Work Package (WP1):** The PRECISION project enables the collection of large tissue resources from both retrospective and prospective clinical trials. Supplementary Table 1 gives an overview of the cohorts which will form the basis of the PRECISION effort. Clinical data, including long-time follow up, tissue blocks and imaging data obtained through pooling multiple retrospective clinical studies based in the USA, UK and the Netherlands, are available for an in-depth characterisation of indolent and aggressive DCIS. These series will be used in WP2, 3 and 4 to gain a deep and thorough understanding of DCIS. Most importantly, to address the heterogeneity issue in DCIS, the studies contain large datasets, collected in different settings (population/hospital based and screening setting) and capture different populations.

***Supplementary Table 1.*** *Sources for DCIS cohort for tissue collection and prospective DCIS trial for watchful waiting.*

| **Retrospective series** |  |  |  |  |
| --- | --- | --- | --- | --- |
| **Series** | Description | Size (total n/ recurrences) | Median follow up (year) | Ref. |
| **Dutch DCIS cohort** | population based cohort 1989-2004 | 10,096/ 1,200 | 11.6 | Elshof et al, 2016^22^ |
| **Sloane project** | UK NHS Breast Screening Prospective cohort | 13,000/800 | 9.2 | Thompson et al, 2018^21^ |
| **MDAnderson series** | US MD Anderson retrospective cohort 2003-2015 | 2.500/200 | 5 | NA |
| **Duke series** | US Duke retrospective cohort | 658/NA | 5 | NA |
| **Prospective clinical trials** |  |  |  |  |
| **Trial acronym** | Description | Size (target number of participants) | Main outcome | Ref. |
| **LORIS** | A trial comparing surgery with active monitoring for low risk DCIS (LORIS) | 900 | Ipsilateral invasive BC-free rate at 10 years | Francis et al, 2015^56^  NCT02766881 |
| **COMET** | Comparison of Operative to Monitoring and Endocrine Therapy (COMET) Trial For Low Risk DCIS (COMET) | 1200 | Proportion of new diagnoses of ipsilateral invasive cancer in GCC and AS arms at 2 years of follow up | NCT02926911^57,58^ |
| **LORD** | Low risk DCIS study | 1240 | Ipsilateral invasive BC-free rate at 10 years | Elshof et al, 2015^59^  NCT02492607 |

**Comprehensive genomic characterisation of DCIS (WP2)**: To determine whether a lesion is life-threatening, a clear understanding of the biology of DCIS is required to identify the critical drivers of DCIS evolution and progression to invasive disease. To identify putative novel drivers, whole genome and whole exome sequencing are performed to identify the mutation spectrum, the sequence of each gene, the impact of coding substitutions (synonymous, missense, nonsense, splice site) and the variation of the mutation rate across genes. The landscape of base substitution mutational signatures in DCIS will be assessed and compared to what is already known for invasive breast cancer. A bank of genomic data is being created for future analysis. A key feature of our genomic studies is to capture both the interpatient and intratumoural heterogeneity. The first is addressed by profiling large sets of samples from various studies (see WP1). The latter is addressed by multiregim sequencing and single cell studies.

**Characterising the role of the immune microenvironment as a determinant of DCIS clinical biology (WP3):** The main hypothesis being explored is that features of the immune microenvironment are key in determining the risk of DCIS progression. The immune microenvironment of DCIS are profiled by multiplex immunohistochemistry and immunofluorescence assays. Furthermore, T-cell receptor clonality and neo-antigen prediction will be investigated.

**Investigating the role of imaging in DCIS prognosis and outcome (WP4)**: The overarching goal is to identify a series of systematic differences in the radiographic and physicochemical characteristics of lethal versus non-lethal cancers captured on images to DCIS biology. The first objective is to develop novel molecular mapping approaches to quantitatively characterise DCIS tissue using mid-infrared absorption and Raman spectroscopic imaging of soft tissue and calcifications. The second objective is to create and test a computational learning algorithm to compare mammographic characteristics and diversity measures in pure DCIS compared to DCIS with IDC. The third objective is to validate the algorithm in PRECISION’s retrospective cohorts (WP1) and in data obtained from the prospective clinical trials (see below; WP7).

**Functional validation of DCIS drivers (WP5):** The aim of this WP entails the functional validation of candidate DCIS genes from the comprehensive genomic characterisation of DCIS samples. To critically assess the functional relevance, reliable animal models are essential. Recent advances in CRISPR/Cas9-based somatic gene editing, three-dimensional (3D) organoid culturing and patient-derived tumour xenografting have resulted in a number of novel approaches that can be applied to *in vitro*/*in vivo* validation of candidate DCIS genes and to *in vitro*/*in vivo* propagation of viable DCIS samples from patients.^94–99^

**Building a clinical risk stratification model (WP6):** All information obtained from the previous steps will be collated to develop a DCIS risk prediction model, integrating all clinical, morphological, molecular and imaging data. The most promising molecular markers will be combined in an easy-to-use clinical assay. The risk prediction model and clinical assay will be validated in the prospective clinical trials (LORIS, COMET and LORD, see below in next section).

**Validation of molecular markers in active surveillance using the LORIS, LORD and COMET trials (WP7):** The LORIS (United Kingdom, NCT02766881)^56^, COMET (United States, NCT02926911)^57^ and LORD (The Netherlands, NCT02492607)^59^ randomised trials together present a unique opportunity. They have a common aim of assessing which low or intermediate grade DCIS requires primary surgical management and whether regular monitoring for disease progression by mammography can be safely performed, with intervention only in those women in whom there is evidence of progression to high-risk DCIS or invasive cancer. The common research objectives of the three trials are to evaluate the safety, effectiveness, cost effectiveness and acceptability of non-surgical intervention in patients with newly diagnosed, mammogram detected asymptomatic, low or low-intermediate grade DCIS; and to define the natural history of low-risk DCIS and to identify those patients who require surgery because their DCIS is at risk of progression to invasive disease.

All the above trials are prospectively randomising patients with screen-detected or incidental low risk of recurrence DCIS to standard surgical treatment or active monitoring. The trials had started before the inception of the PRECISION initiative, but the initiative gave us a unique opportunity to collaborate, and to safeguard tissue and blood collections for translational biomarker research. LORD, LORIS and COMET are recruiting and expect to complete recruitment within 5 years. In addition to collaborating and exchanging valuable information regarding accrual and patient participation, we plan to assess the value of circulating tumour DNA and genomics approaches in blood and tissue samples of trial participants.

**
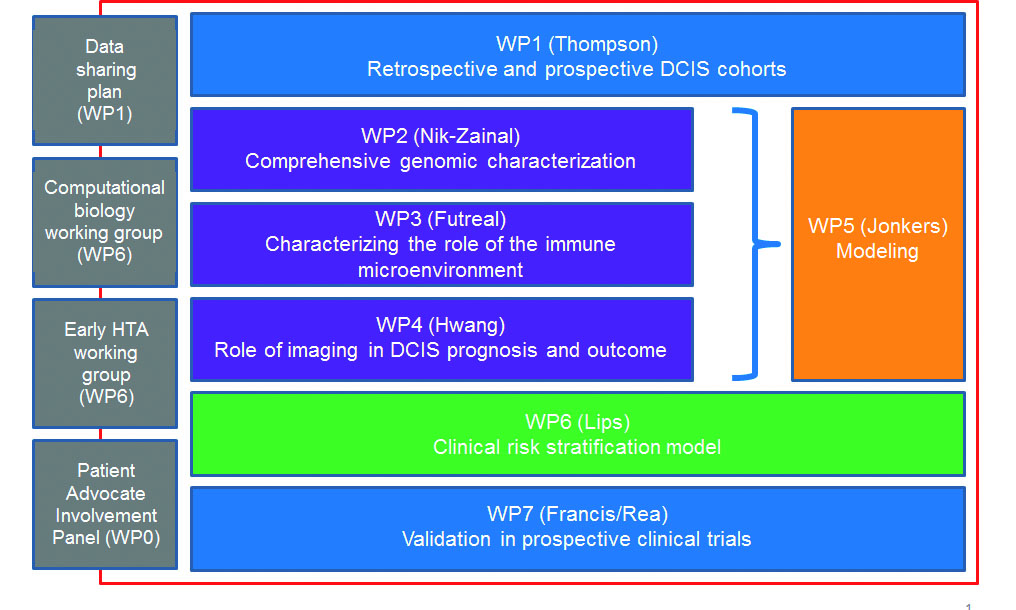
**

***Supplementary Figure 1****. Overview and links to the different work packages (WP) within the PRECISION project*

**Patient involvement**

International expert patient advocates with previous experience of DCIS, cancer or another condition are centrally involved in PRECISION. Their contributions are at three levels: project governance; scientific work; and outreach to the general public, patients and physicians, which helps to reshape clinical practice and the public perception of DCIS risk. The scope and depth of patient involvement is possible because all patient advocates are highly experienced and knowledgeable about the attitudes of their respective countries toward DCIS. Each patient advocate adds professional patient expertise combined with personal experience to the science that will be conducted in PRECISION. They are also directly involved with the prospective LORD, LORIS, and COMET trials in each country, and can relate to the long-term quality of life issues that women face from current DCIS treatments. Their goal is to replace fear of DCIS with confidence that each woman will receive effective, evidence-based treatment (or monitoring alone) that matches her specific type of DCIS, based on personalized invasive cancer risk that PRECISION will help to elucidate.
